# Outbreak Control and Clinical, Pathological, and Epidemiological Aspects and Molecular Characterization of a Bovine Herpesvirus Type 5 on a Feedlot Farm in São Paulo State

**DOI:** 10.1155/2015/981230

**Published:** 2015-05-21

**Authors:** Jane Megid, Acácia Ferreira Vicente, Camila Michele Appolinario, Susan Dora Allendorf, Mateus de Souza Ribeiro Mioni, Thaís Gasparini Baraldi, Adriana Cortez, Marcos Bryan Heinemann, Clovis Reinaldo Silva Fonseca, Vanessa Cristina Pelícia, Bruna Leticia Devidé Ribeiro, Liria Hiromi Okuda, Edviges Maristela Pituco

**Affiliations:** ^1^Universidade Estadual Paulista Julio de Mesquita Filho (UNESP), Faculdade de Medicina Veterinaria e Zootecnia, Departamento de Higiene Veterinária e Saude Publica, 18618-970 Botucatu, SP, Brazil; ^2^Universidade de Santo Amaro (UNISA), Faculdade de Medicina Veterinária, 04829-300 São Paulo, SP, Brazil; ^3^Universidade de São Paulo (USP), Faculdade de Medicina Veterinária e Zootecnia, Departamento de Medicina Veterinária Preventiva e Saúde Animal, 05508-270 São Paulo, SP, Brazil; ^4^Instituto Biológico de São Paulo, Laboratório de Viroses dos Bovídeos, São Paulo, SP, Brazil

## Abstract

This paper describes the control, epidemiological, pathological, and molecular aspects of an outbreak of meningoencephalitis in calves due to bovine herpesvirus 5 at a feedlot with 540 animals in São Paulo State, Brazil. The introduction of new animals and contact between the resident animals and the introduced ones were most likely responsible for virus transmission. Bovine herpesvirus 1 vaccine was used, resulting in the efficacy of the outbreak control, although two bovine herpesvirus 1 positive animals, vaccinated and revaccinated, presented meningoencephalitis, thereby characterizing vaccinal failure.

## 1. Introduction

Bovine herpesvirus type 5 (BoHV-5) belongs to the family Herpesviridae, subfamily Alphaherpesvirinae, and genus* Varicellovirus* [1] and is the etiological agent of nonsuppurative meningoencephalitis, which has been described worldwide but has a higher occurrence in Latin America [2]. Outbreaks of meningoencephalitis caused by BoHV-5 have been reported mainly in South American countries such as Argentina [3, 4] and Uruguay [5]. BoHV-5 outbreaks are sporadic and appear to be restricted in their geographical distribution. The reason for the discrepancy in the prevalence between South America and the rest of the world is unknown. The absence of a specific tool for the detection and identification in vivo of BoHV-5 infected animals does not allow differentiation between BoHV-1 and/or BoHV-5 positive animals; therefore, the true prevalence of the infection is unknown and the economic relevance remains to be determined [2]. In Brazil, the disease has been reported mainly in Rio Grande do Sul [6–8] and less frequently in other states such as Mato Grosso do Sul [9], São Paulo [9], Paraná [10], Mato Grosso [11], Rio de Janeiro, Minas Gerais [12], and Pará [13]. Epidemiological aspects that increase virus dissemination include the aggregation of animals, introduction of animals from other places, and stressful situations [14]. The respiratory tract is a replication site for BoHV-5, and viral shedding in nasal secretions constitutes an efficient source of transmission during acute infection. BoHV-5 infection can induce a subclinical infection or mild disease in adult cattle [15, 16], but, in young animals, it frequently causes meningoencephalitis, reaching 75–100% mortality [13, 17].

The aim of this work is to describe the clinical, epidemiological, and molecular characterization of the BoHV-5 associated with an outbreak of meningoencephalitis at a feedlot farm in São Paulo State.

## 2. Case Report

The farm manager reported the introduction of new animals to the intensive system of the farm in March 2013. The farm is characterized by the constant acquisition and introduction of animals from different regions. These purchased animals were kept in paddocks for a period of 3 months for adaptation being transferred to the feedlot. The farm had a total of 540 animals and the outbreak, never reported in the past, was observed in three different paddocks where more or less than 130 animals were confined together. These paddocks were divided by fences allowing the respiratory transmission. One month later, two animals (12 months old) that had been introduced to the farm started to present clinical signs of isolation, cessation of drinking and eating, abundant salivation, loss of vision, disorientation, nasal discharge, incoordination, walking in circles, and seizures that were sustained for approximately two minutes before the animals recuperated and stood again. The clinical course was approximately 7 days. The appearance of the disease occurred within one day of each other for the two animals that first showed symptoms. There was a lack of diagnosis for these cases. Six months later, animals that were born on the farm were introduced into the same pasture where the disease and deaths were observed previously and entered into contact with animals that had also stayed in this pasture during the first cases (some of the introduced animals had been placed together with resident animals by accident). One month after the introduction of these animals, the same clinical signs were observed in 9 resident cattle, 12 months old, with clinical onset ranging from over one to two weeks and the outbreak occurring over a period of two months. All cases occurred in the same paddocks.

## 3. Material and Methods

Three of these animals were brought to the Infectious Diseases of Domestic Animals Service at Faculdade de Medicina Veterinária e Zootecnia de Botucatu, UNESP. Two animals underwent a clinical examination, which included the collection of blood, biochemical samples, and cisternal cerebrospinal fluid (CSF). Necropsies were performed on all three animals after natural death and carcasses were incinerated afterwards. Brain and lung from two animals were analyzed by histopathology. Brain, CSF, and serosanguinous fluid consequent to cerebral edema and lung from 3 animals were submitted to PCR. For one animal, in addition to these samples, spinal cord, submandibular and mediastinal lymph nodes, liver, spleen, kidney, testicles, blood, saliva, and a nasal swab were also submitted to a nested PCR using primers PF (GTG GAG CGC CGC TTC GC) and PR (TAT CGC GGA GAG CAG GCG) [18] for BoHV-1 and BoHV-5 differentiation. The brains from the positive samples were analyzed by real-time PCR using primers BoHV F (TAC GGA CTG CCG GAT TAA CA) and BoHV R (GTC ACC CTA CCA CCG CCG CCA ACA T), according to the protocol of Pedraza-Ordoñez et al. [19]. Amplicons were purified from agarose gel using a commercial kit (GE Illustra GFX PCR DNA and Gel Band Purification Kit). After determining the nucleotide sequences of the amplified reaction using real-time PCR that targeted the glycoprotein G region, sequencing reaction fragments were subjected to the PHRED (http://www.biomol.unb.br/phph/index.html) computer program for quality analysis of the chromatograms. As a criterion for quality validation, only readings that were greater than or equal to a score of 20 were accepted.

To verify the identity of the sequences generated, a search was conducted at GenBank database sequences using Blast (http://blast.ncbi.nlm.nih.gov/Blast.cgi?PROGRAM=blastn&PAGE_TYPE=BlastSearch&LINK_LOC=blasthome).

The Bot01, Bot02, and Bot03 sequences were aligned using the ClustalW program within the computational package BioEdit [20], which led to the glycoprotein G gene available in GenBank Brazilian sequences. The accession numbers for the applicable sequences included AF 330157, AF 298174, and AY 916518 from bovine semen; the 840 481, 840 482 DQ, and DQ 840 483 for respiratory symptoms in cattle; and 261 359 AY, AY 916517, AF 298175, AF 330158, and AF 366571 from the CNS. Using this information, an identity matrix was constructed. An analysis of the genetic relations of the sequences was performed with the aid of the MEGA 6.0 computer program [21] with the evolution model, which compared the maximum likelihood composite samples of bovine herpesvirus type 5 and the above with a sample of bovine herpesvirus type 1 (AY242113).

## 4. Results

**Figure 1:**
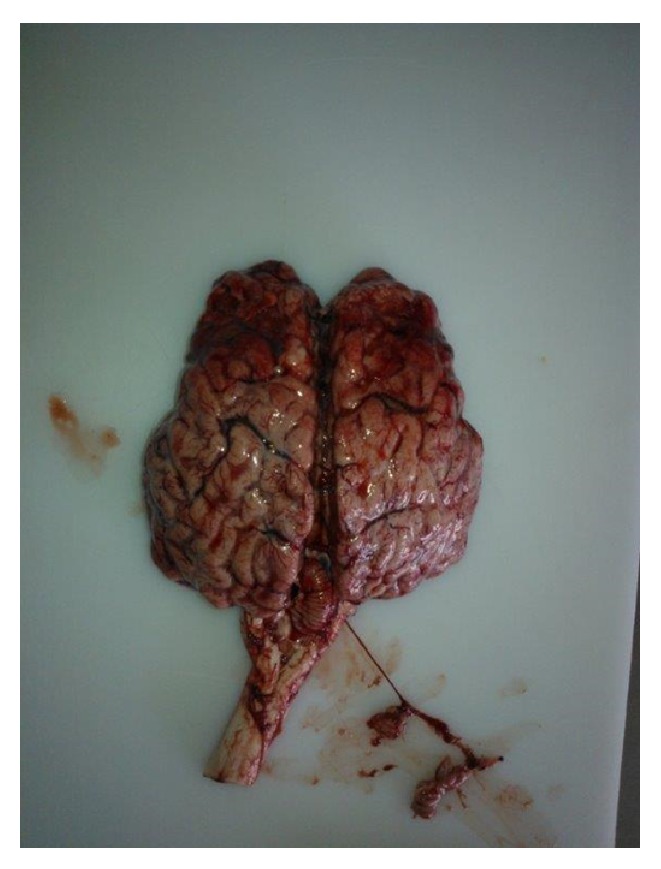
Brain haemorrhage, edema, and softening of the parenchymal tissue in the frontal area.

Clinical signs were characterized by serous nasal secretion, blindness, incoordination, hyperexcitability, mouth myoclonus in one animal, seizures, external recumbency, and death occurring in 1 to 3 days. Blood analysis were performed in two of the cases, and this analysis showed leukocytosis with neutrophilia, lymphocytosis, and monocytosis; serum protein, electrolytes, creatine kinase (CK), urea, and globulin were high in both animals; and GGT (gamma glutamyltransferase), AST (aspartate aminotransferase), and creatinine were also elevated in one of the animals. Cerebrospinal fluid (CSF) from the animals showed relatively high levels of protein (134.5/154.3 mg/dL), glucose (+/++), positive Pandy test demonstrating the presence of globulins, pleocytosis with mononuclear cells predominance (270/153), red cells (2640/72), and xanthochromia in one of the animals. Macroscopic lesions from the lung demonstrated emphysema and petechial hemorrhage; histopathological evaluation showed focal hemorrhage, alveolar and interstitial emphysema, marked edema, and the presence of fibrin in the alveoli, as well as interstitial mixed inflammatory infiltrate (interstitial pneumonia). In the brain hemorrhage and edema, meningeal thickness and softening of the parenchymal tissue in the frontal area (Figure 1) were observed macroscopically. Histopathology from the brain and spinal cord showed congestion, edema, multifocal necrosis in gray matter, and mononuclear perifocal cuffing were also observed. The cerebellum presented congestion, edema, mononuclear perivascular cuffing, lymphocytes in the meninges and white matter, chromatolysis of Purkinje cells, and disseminated hemorrhage. Lesions on the cerebral cortex were characterized by congestion, edema (dilatation of Virchow-Robin space), perivascular cuffing of the monocytes and lymphocytes, multifocal necrosis of neuron bodies, multifocal hemorrhage, and focal areas of polioencephalomalacia. Enteritis and reactivity in the mesenteric lymph nodes were visualized at the necropsy. Brain tissues were submitted for rabies, thromboembolic meningoencephalitis, BHV-1 and other bacterial agents differential diagnosis resulting negatives. Polymerase chain reaction (PCR) was used to check BoHV-5 in samples from the three calves and it was positive for only the brain and serosanguineous cerebral fluid with negative results for the lung samples. In one animal, the spinal cord and submandibular lymph node were also evaluated according to positive PCR results. In two animals, serosanguineous cerebral fluid was collected and was positive for BoHV-5 according to PCR. Virus isolation was obtained from brain samples, and the cytopathic effect was observed. Considering the positive result in PCR, the remaining animals were vaccinated with two doses (one month apart) of multiple vaccines containing attenuated BoHV-1. Measures were also undertaken to restrict animal movement on the farm, and environmental disinfection was carried out where possible. No additional deaths were observed on the farm for 4 months; then, one animal already vaccinated and revaccinated presented the same clinical signs. This animal (18 months of age) was also brought to our clinical service showing the same signs as the first animals, but the evolution period was longer. The animal was euthanized in the agonic stage for humanitarian reasons. Necropsy was performed, and similar macroscopic lesions were observed. BoHV-5 PCR resulted positive for brain and spinal cord samples. Three months later, another death in a vaccinated and revaccinated animal that showed the same clinical signs and suggested the same diagnosis (this animal was not attended by our service) was reported by the farm manager. The manager also reported that another vaccinated animal had displayed the same clinical signs but had recovered from the disease and was still being maintained on the farm (contrary to veterinary recommendations).

After the quality of sequences generated for Bot01, Bot02, and Bot03 were analyzed, a search for similar sequences was performed in the GenBank database, and those recovered indicated only the G glycoprotein of BoHV-5. Subsequent alignment of the sequences resulted in a fragment of 205 bp and gave an identity matrix with full identity among the Bot01, Bot02, and Bot03 samples and among the AY 261359, AF 298175, and AY 916517 samples from the central nervous system of cattle and the AF 330157, AF 298174, and AY 916518 samples that were derived from bovine semen. Furthermore, 1 amino acid difference was found when the identity matrix was compared to the AF 330158 and AF 366571 samples, which also came from the CNS. Samples derived from a respiratory outbreak and found in coinfection with respiratory syncytial virus showed that 3 amino acids were different, compared to the samples that exhibited complete identity (DO 840 481, 840 482 DQ, and DQ 840 483).

**Figure 2:**
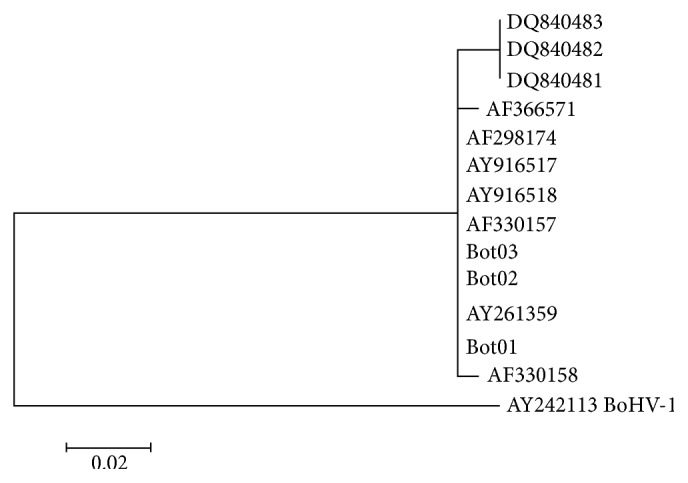
Cladogram for the G glycoprotein gene obtained with the Brazilian samples available in GenBank for BoHV-5 and Bot01, Bot02, and Bot03 samples and outgroups using BoHV-1.

The cladogram (Figure 2) shows the formation of two groups, one corresponding to samples of herpesvirus type 5 and the other corresponding to bovine herpesvirus type 1, corroborating the above data, which indicate that Bot01, Bot02, and Bot03 fragments are derived from samples of BoHV-5.

## 5. Discussion and Conclusion

Epidemiological aspects suggest that the introduction of infected animals into a new place, which creates a stressful situation, was the factor responsible for triggering disease in the first two animals, and this disease was characterized by an incubation period of approximately 30 days and an evolution period of 7 days, similar to those observed in other reports [9, 13, 22]. These animals were in a paddock with other introduced animals that did not present the disease but most likely had latent infections. It is probable that when the resident animals were placed in this paddock six months later, they had contact with latent infected animals. These latent infected animals then transmitted the virus, starting an outbreak among the animals that were not immunized against herpesvirus and were placed in a stressful situation [2, 13, 17].

### 5.1. Blood Cells, CSF, and Clinical Biochemistry Analyses

Leukocytosis with neutrophilia, monocytosis, and lymphocytosis, characterizing a lymphoproliferative response, then occurred 7–14 days after infection [2]. Elevated levels of urea, electrolytes, and serum protein were observed and can be explained by dehydration due to anorexia. Higher levels of AST and CK were observed experimentally in infected animals with bovine spongiform encephalopathy [23]. The authors considered these enzymes helpful for diagnosing muscle injury, but they may not provide information concerning the origin (myopathy or neuropathy) of the problem. In this paper, authors associated high levels of enzymes with muscular damage, but, in the present report, affected animals did not show muscle lesions despite the high levels of CK and AST. Alterations observed in CSF analysis confirmed an inflammatory process in the brain, and these alterations were similar to those observed by other reports [24], which were characterized by pleocytosis that was predominantly mononuclear, indicating viral meningoencephalitis [25].

### 5.2. Histopathology

Macroscopic and histopathological lesions were similar to those in previous reports, but inclusion bodies were not observed [8, 9, 13, 26].

### 5.3. Molecular Biology

In the present report, BoHV-5 was detected predominantly in the brain and cerebral serum fluid in accordance with several reports [2, 8, 9, 13, 14, 24, 26, 27]. Although BoHV-5 replicates in the respiratory system and produces high titers of virus that are shed in the nasal secretions and this process is considered to be responsible for virus transmission during acute infection [2, 16], no virus was detected in the lungs of the three calves nor in the saliva or on the nasal swab from the one calf, even while these animals were in the acute phase of the disease. These results are in accordance with Cascio et al. [15] who report low virus titers in respiratory secretions. However, positivity was observed in a submandibular lymph node from one animal, suggesting a latency site for BoHV-5 similar to that reported by Mweene et al. [28], who detected the virus in a prescapular lymph node.

The BoHV-5 virus detected in this outbreak from the four affected animals that were attended by our service was sequenced and showed to be similar to the BoHV-5 characterized in Brazil, demonstrating the wide dissemination of this virus in our country.

### 5.4. Cattle Immunization

Considering that no specific BoHV-5 vaccine is commercially available in Brazil, immunization with BoHV-1 aims for protection against BoHV-5 in areas where the disease has been diagnosed as common [9]. Cross protection between BoHV-1 and BoHV-5 has been reported, as well as the efficacy of BoHV-1 vaccine against BoHV-5 infection in BoHV-1 vaccinated cattle (two doses, 0 and 21 days of inactivated vaccine) that were experimentally challenged with BoHV-5 virus [16]. The authors reported humoral and cellular immune response with high levels of protection and with only slight neurological symptoms and intermittent virus excretion in one vaccinated animal; thus, they concluded that the BoHV-1 vaccinated animals were protected against neurological disease caused by BoHV-5. In Brazil, only one recombinant BoHV-1 gE deleted vaccine was tested against BoHV-5, and it presented limited efficacy. The authors reported that BoHV-5 nasal shedding was not reduced and that there was no protection against brain lesions in vaccinated animals. However, there was a reduction in the intensity of clinical signs after primary infection [29]. A decrease in virus shedding, as well as a reduction in the period of virus elimination, was reported in animals vaccinated with BoHV-1 traditional vaccines [15]. Attempts to reactivate the virus with dexamethasone in BoHV-1 vaccinated and BoHV-5 challenged animals did not result in respiratory or nervous symptoms in calves despite the presence of BoHV-5 virus in their nasal secretions [30].

In our paper, the disease was controlled on this farm with 540 animals after two doses of BoHV-1 vaccination in all animals, thereby demonstrating herd protection. Although two previously BoHV-1 vaccinated and revaccinated cattle presented nervous clinical signs, one with a longer incubation period followed by death and another animal that survived, this seems to be more suggestive of vaccine failure than virus reactivation from the previously infected animals.
